# An Eye into the Aorta: The Role of Extracellular Matrix Regulatory Genes *ZNF469* and *PRDM5*, from Their Previous Association with Brittle Cornea Syndrome to Their Novel Association with Aortic and Arterial Aneurysmal Diseases

**DOI:** 10.3390/ijms25115848

**Published:** 2024-05-28

**Authors:** Peyton Moore, Adam Wolf, Mohanakrishnan Sathyamoorthy

**Affiliations:** 1 Sathyamoorthy Laboratory, Department of Medicine, Burnett School of Medicine at TCU, Fort Worth, TX 76104, USA; 2 Consultants in Cardiovascular Medicine and Science, Fort Worth, TX 76104, USA; 3 Fort Worth Institute for Molecular Medicine and Genomics Research, Fort Worth, TX 76104, USA

**Keywords:** *ZNF469*, *PRDM5*, extracellular matrix, aortic aneurysm, genetic basis of aortic disease, corneal diseases

## Abstract

The extracellular matrix is a complex network of proteins and other molecules that are essential for the support, integrity, and structure of cells and tissues within the human body. The genes *ZNF469* and *PRDM5* each produce extracellular-matrix-related proteins that, when mutated, have been shown to result in the development of brittle cornea syndrome. This dysfunction results from aberrant protein function resulting in extracellular matrix disruption. Our group recently identified and published the first known associations between variants in these genes and aortic/arterial aneurysms and dissection diseases. This paper delineates the proposed effects of mutated *ZNF469* and *PRDM5* on various essential extracellular matrix components, including various collagens, TGF-B, clusterin, thrombospondin, and HAPLN-1, and reviews our recent reports associating single-nucleotide variants to these genes’ development of aneurysmal and dissection diseases.

## 1. Introduction

The extracellular matrix (ECM) is a network of proteins and other molecules that are essential for support, integrity, and structure for tissues and cells within the body. Though essential in various tissues, the ECM is particularly a critical player in vascular integrity. Through interactions with endothelial cells, the ECM provides scaffolding that is important for blood vessel development and stabilization [1]. The arterial ECM is composed of various molecules, including elastin, collagen, proteoglycans, and glycoproteins. The vascular ECM undergoes continuous remodeling, consisting of protein synthesis and the replacement of matrix proteins throughout the tunica intima, media, and adventitial layers. Errors in the physiologic remodeling of the aortic wall are responsible for aortic aneurysm development, which is characterized by irreversible dilatation of the aortic lumen by greater than 50 percent of its original diameter [2]. A genetic influence of thoracic aortic aneurysm and dissection (TAAD) development is apparent, as approximately 30 genetic variations have been identified in patients with TAADs. Many of these known variations have a significant role in ECM development and maintenance, further suggesting a critical role between ECM regulation and TAAD development [3].

The ECM is also a vital component of ocular development and integrity, as it provides a meshwork for cellular structure. Components of the ocular ECM include proteoglycans, collagen, elastin, laminin, fibronectin, fibrillin, and hyaluronic acid, among various other extracellular proteins [4]. ECMs are present within various compartments of the eye, particularly the stroma of the cornea. The corneal stroma is responsible for 90% of corneal thickness and has been identified as a densely packed, collagen-rich ECM [5]. Defects in ECM genes and their subsequent regulators have resulted in ocular disease, particularly in syndromes related to the ECM-rich cornea [6].

There is a continuing interest in evaluating variants in genes related to ECM development, maintenance, and physiologic remodeling, as several have shown to result in TAAD phenotypes. Our group has recently identified that two genes, *ZNF469* and *PRDM5*, previously only associated only with brittle cornea syndrome (BCS), are now associated with the development of aortic and arterial aneurysmal diseases (TAADs). This review provides an au courant association of known ECM regulatory genes *ZNF469* and *PRDM5* in the development of ECM-related diseases BCS and TAAD, establishing a potential connection between aneurysmal diseases and corneal ocular disease.

## 2. Brittle Cornea Syndrome and Its Association with *ZNF469* and *PRDM5*

### 2.1. Brittle Cornea Syndrome

Brittle cornea syndrome (BCS) is an autosomal recessive connective tissue disorder characterized by ocular and extra-ocular findings. Common ocular features of the disorder include thinning of the cornea, keratoconus, keratoglobus, and myopia. Corneal thinning, the hallmark of the disorder, increases the risk of corneal rupture in these patients, with a potential for irreversible blindness [7]. Corneal rupture is the most frequent presenting feature of BCS, often from spontaneous rupture or due to minor injury [8]. Extra-ocular manifestations of BCS include hyperlaxity of the skin and joint hypermobility, two common findings in generalized connective tissue disorders [9,10,11,12].

Development of BCS is a result of a gene variant in one of two genes: *ZNF469* and *PRDM5* [7]. Though the exact mechanism of ECM regulation is unknown for both *PRDM5* and *ZNF469*, a comparable downregulation of ECM-associated genes was observed in mutants, suggesting a common pathway for both [6]. Similar molecular changes in ECM genes and regulators with variants in *ZNF469* and *PRDM5* results in phenotypes without significant variation from each other, further suggesting a common regulatory pathway.

While these genes have been directly attributed to the ocular manifestations of BCS, variants in both have also resulted in extra-ocular symptoms. These symptoms, often characteristic of generalized connective tissue disorders, increase the suspicion of their involvement in other disease processes that ECM-related variants are responsible for, including aortopathy.

### 2.2. ZNF469

**Figure 1:**
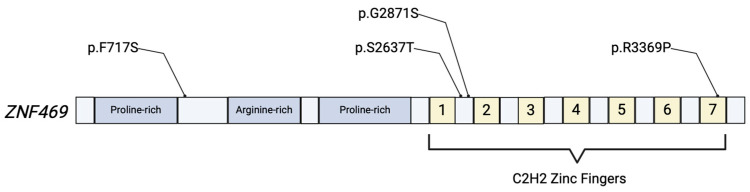
Simplified illustration of the ZNF469 protein product. The protein includes proline-rich and arginine-rich domains, and 7 C2H2 zinc fingers that are responsible for the proteins’ role in regulation of gene expression. Various mutations have been identified in ECM-related disease, with a few depicted above [6,17].

Zinc-Finger Protein 469 is a collagen-related protein encoded by the poorly understood gene *ZNF469*, located on chromosome 16q24.2 (Figure 1). There is a shared homology of 30% between the ZNF469 protein with clusterin and collagens I and III, strongly suggesting it plays a part in the production or regulation of collagen fibers [13]. This gene is a member of the zinc-finger gene family, one of the most common motifs in eukaryotes. Zinc-finger motifs typically interact with DNA/RNA, making them important in the regulation of gene expression. ZNF469 was inferred to regulate the transcription of 26 s proteasome family members, thereby promoting the degradation of the ECM, as well as regulating the transcription of ECM component genes [14]. Stanton et al. demonstrated that, through ECM disruption, ZNF-knockout mice experienced a significant decrease in biomechanical strength of the cornea [15]. Certain variants in *ZNF469* have been identified as pathogenic for BCS, and novel variants continue to be discovered [6,16].

### 2.3. PRDM5

**Figure 2:**
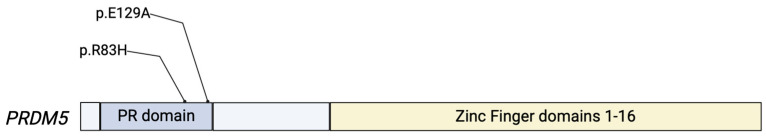
Simplified protein structure of the PRDM5 protein. The protein contains an intact PR domain at the NH-terminus, with 16 zinc-finger domains near the C terminus. The PR- and zinc-finger-domains are responsible for the proteins’ role in transcriptional regulation. Two variants in the *PRDM5* gene that have been reported in prior ECM-related diseases are shown [17].

The *PRDM5* gene is located on chromosome 4q27 and is a 17-exon protein-coding gene responsible for production of the PRDM5 protein (Figure 2). The PRDM5 protein is recognized as a transcription factor of the PR-domain protein family, which contains a PR-domain and multiple zinc-finger motifs [13]. The functional transcription factor is involved in tumor suppression and the regulation of extracellular matrix development in corneal and bone cells [18]. PRDM5 as a transcription factor is crucial for both ECM development and maintenance in the cornea [8]. Variants in *PRDM5* in humans have been associated with BCS development, with a disruption in its role as an ECM regulator being the suggested mechanism for pathogenesis [7]. In subjects with variants in *PRDM5*, the downregulation of genes that encode molecules such as fibrillar collagens, connective tissue components, and various molecules involved in cellular migration and adhesion regulation was observed on a microarray analysis of dermal fibroblasts. The variant resulted in an increased corneal fragility and decreased corneal thickness [8].

## 3. ECM Genes in Thoracic Aortic and Aneurysmal Dissection Disorders (TAADs)

**Figure 3:**
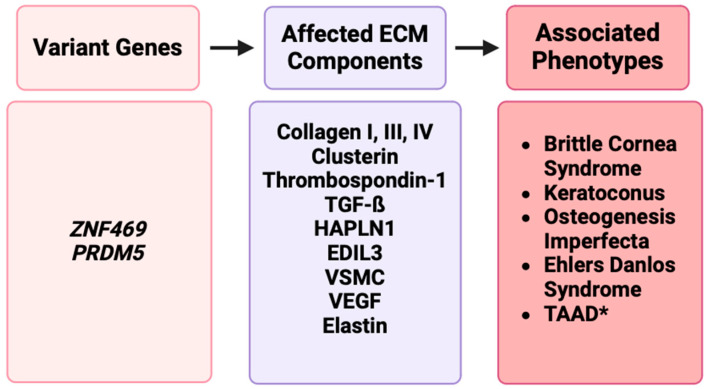
A representation of disruptions in ECM genes such as *ZNF469* and *PRDM5* affecting ECM components such as collagens, clusterin, elastin, or other proteins, resulting in phenotypes such as BCS, keratoconus, osteogenesis imperfecta, Ehlers–Danlos syndrome, and TAAD [17].

Of the known ECM genetic variants that are classically indicated in the development of TAADs, there are three major categories: collagen disruptors, elastic fiber disruptors, and transforming growth factor-beta (TGF-β) disruptors [19]. Further, genes related to smooth muscle cell (SMC) contractility have been implicated in TAAD development. These categories are natural candidates, given that collagen and elastin are major constituents of the human aorta, while TGF-β signaling is a key player in the function and maintenance of the aortic wall. The aorta, and specifically the thoracic segment, is under great pressure and, as such, the maintenance of function is of the utmost importance and any compromise can have extreme consequences. To achieve that stability, it is important that each of the integral components of the aortic wall are functioning properly. Disruptions in genes such as PRDM5, ZNF469, collagen-encoding genes, or other genes responsible for ECM development and/or integrity may predispose individuals to ECM-related diseases (Figure 3).

### 3.1. COL Genes

In arterial vessels, the dominant types of collagen present are I (heterotrimer) and III (homotrimer), which are encoded by *COL1A1/2* and *COL3A1*. These collagens are chiefly responsible for the aortic walls’ tensile strength via a triple-helix configuration [2]. Though the composition varies person-to-person, studies have shown that the concentration of collagen in the aortic wall generally increases significantly with age and comprises around ~25% of the thoracic aorta [20,21]. *COL4A1/2* produces collagen IV, which is less abundant overall but is prominent in the basement membranes of endothelial cells and intimal/medial smooth muscle cells [22].

Variants in *COL1A1* and *COL1A2*, especially glycine-related missense variants, are classically associated with osteogenesis imperfecta (OI), a heritable disorder characterized by bone fractures, hearing loss, and dental impairments. Though a rarer effect, there have been reports of OI family cohorts with aortopathy [23]. There have also been reported cases of variants replacing y-position arginine with glycine or cystine, leading to aneurysmal disease [24]. Variants in *COL3A1* are associated with vascular Ehlers–Danlos syndrome (vEDS) and have a wide array of devastating vascular consequences. Collagen IV has been identified as a protective agent against the development of abdominal aortic aneurysms (AAAs) by Steffensen et al., who developed a murine model showing knockout mice with a deficiency in *COL4A1/2* correlated with AAA progression, in addition to a Danish human cohort demonstrating that increasing collagen IV degradation product levels correlate with the progression of AAAs [25]. Due to its important role in maintaining the function and integrity of the ECM, disruptions in collagen are associated with numerous ECM-related diseases, including TAAD.

### 3.2. Transforming Growth Factor-Beta (TGF-β)

TGF-β signaling plays a critical role in vasculature development and maintenance. Variants in *TGFB1/2* have been identified in several syndromic causes of thoracic aortic aneurysm and dissection (TAAD), including Marfan syndrome (MFS), Loeys–Dietz syndrome (LDS), and Shprintzen–Goldberg syndrome (SGS). MFS is classically associated with pectus deformity, myopia, valvular regurgitation, arachnodactyly, and, due to cystic medial necrosis (CMN), aortic aneurysm. The role of TGF-β in the formation of TAAD has been a controversial topic in the literature, with many studies showing conflicting results. One side of research has demonstrated a pathogenic role for TGF-β, with dysregulation of TGF-β signaling due to variants in fibrillin-1 (*FBN1*) being contributory to CMN of the medial layer in the aortic wall, leading to aneurysm development [26]. In this, functional FBN1 binds TGF-β ligands, and a loss of FBN1 is hypothesized to lead to an increase in bioavailable TGF-β. Dysregulated signaling due to an increased availability in TGF-β is partially responsible for aneurysm development [27].

Other research has shown a dimorphic effect for TGF-β in TAAD formation, with an emphasis on the contextual state of the disease. In the early stage of TAAD formation, TGF-β was shown to be beneficial and a physiological response to a structurally compromised aortic wall under stress. In the late stages of TAAD formation, TGF-β was determined to be pathogenic. This was hypothesized to be due to a negative feedback loop that involves immune-inflammatory responses [28].

Further research has found pathogenic variants in TGF-β receptors responsible for the development of LDS, leading to aortic aneurysm development at an early age [26]. Variants in TGF-β receptor 1 (*TGFBR1*) and 2 (*TGFBR2*) have been associated with the development of LDS, a disease that shares many characteristics with MFS [27]. Similarly, increased TGF-β signaling in SGS has negatively impacted aortic integrity. Variants in the SKI proto-oncogene encoding a TGF-β repressor, causing SGS, is responsible for the increase in TGF-β signaling, leading to eventual aneurysm development [26]. Though TAAD development is a common phenotype in SGS, aneurysm disease is commonly less aggressive than LDS [27]. Variants in *PRDM5* have resulted in the downregulation of many important ECM components in fibroblasts, including TGFB2 [8].

## 4. ZNF469

### 4.1. ZNF469 and Association with Arterial Aneurysmal Diseases

To date, we have identified nine patients with aneurysmal or dissection disease with SNVs in *ZNF469*, with many of these patients having one or more first-degree relative with similar vascular phenotypes [29]. These patients, as will be described, are older at presentation when compared to patients with MFS or LDS. The reasoning behind this age presentation discrepancy is unknown but may be worth future research considerations. Our first report described a 72-year-old female who developed chronic bilateral aortoiliac dissections and a subsequent rapidly enlarging aortic aneurysm that underwent operative management [30]. Clinical genotyping revealed three SNVs on Exon 2 of *ZNF469*. A Grantham score was calculated for the three SNVs to calculate evolutionary distance and thus predict the effect of amino acid substitution on the protein product. This score was variable in the three variants, and each was expected to be potentially tolerated by in silico analysis. Though causation probability is not entirely possible to predict in this small sample size, the mutations were thought to disrupt collagen via disturbances in polarity and hydrophilicity and changes in secondary structure propensity [30]. Our subsequent report of our entire cohort of nine patients was notable for a statistical difference in exon localization of the variant and the age of onset/identification of disease in patients. Five patients had variants in exon 1, with an average age of 49, while the four patients with variants in exon 2 had an age of onset/identification of 68 years [29]. One of these patients had a pathogenic variant in *ZNF469* known to be causal for BCS, and though there have been no corneal manifestations of the disease, the patient has fusiform ectasia of the aorta, hypermobility of joints, and a marfanoid habitus. The role of *ZNF469* in ECM regulation is postulated by our group to be the cause of these TAAD phenotypes in our cohorts, with a molecular mechanism similar to the more well-established pathogenesis of BCS. Our group found it interesting that many patients strictly had TAAD phenotypes without signs of BCS. This observation may be due to incomplete penetrance in the variants, or potentially secondary to the location of the variants in the *ZNF469* exon.

### 4.2. COL1A1, COL3A1, and COL4A1 as Regulatory Targets of ZNF469

Given its significant homology, *ZNF469* is predicted to act as a regulator of collagen fiber production. Current research shows that variants in *ZNF469* can disrupt collagen assembly in multiple ways, including collagen receptor dysfunction, decreased collagen IV production via COL4A1 downregulation, and collagen fibril thinning [6,8,31]. Burkitt Wright et al. demonstrated that both *ZNF469* and *PRDM5* variants on patient-derived fibroblasts resulted in a dysregulated/absent type 3 collagen matrix, implying a regulatory role of these genes in the transcription of *COL3A1* [8]. In 2012, Al-owain et al. reported on a large family that had cardinal ocular symptoms of BCS and tested positive for a novel variant in exon 2 of *ZNF469*, and the team noted that the family also possessed EDS phenotypes of severe kyphoscoliosis and joint hypermobility [32]. In addition to EDS-like phenotypes, Rolvien et al. discovered novel *ZNF469* variants in two siblings that had mild BCS that possessed both EDS-like and OI-like phenotypes with blue sclerae, sensorineural and conductive hearing loss, and joint hypermobility [33]. These cases of familial variants in *ZNF469* highlight the overlap between BCS and other collagen-related disorders. These gene variants and associated phenotypes also strengthen the prediction of a relationship between *ZNF469* and *COL* genes.

### 4.3. Clusterin as a Regulatory Target of ZNF469

Clusterin, also known as apolipoprotein J, is a diversely functioning glycoprotein that has been implicated in the development of many diseases, ranging from cerebral amyloid angiopathy to cancer. This chaperone protein aids in the clearance of ECM debris and degradation products and helps to facilitate cell–matrix functions [34,35]. It has been shown to play an important role in vascular smooth muscle cell (VSMC) differentiation and nodule formation [36]. In a mouse model, Shirasawa et al. showed that clusterin-deficient mice experienced a significant reduction in neointimal hyperplasia after induced vascular insult through VSMC cell cycle arrest and the blunting of VSMC proliferation [37]. The clusterin gene (CLU) was found to be significantly altered in ZNF469 mutants and was declared a protein of interest due to its role in ECM interactions and ECM debris clearance [6]. The elucidation of the impact of the ZNF469 variant on clusterin and predisposition to TAAD development warrants further investigation. As stated previously, genes related to SMC contractility have previously been implicated in TAAD development. Due to its role in VSMC differentiation, it is reasonable to suggest that disruptions in *CLU*, particularly due to a variant in *ZNF469*, may lead to TAAD development.

### 4.4. Thrombospondin-1 as a Regulatory Target of ZNF469

The thrombospondin-1 (*THBS1*) gene encodes for an anti-angiogenic glycoprotein known to interact with many ECM components, including elastin [38]. The glycoprotein product TSP1 has demonstrated the ability to bind to and inhibit the processing of the precursor form of the collagen cross-linking enzyme lysyl oxidase. TSP1 was also found to bind to collagen molecules intracellularly, and, when blocked from this function, fibroblasts underwent excessive conversion to myofibroblasts [39]. Though the mechanism remains unclear, this demonstrates a potential for an ECM regulatory responsibility for the TSP1 protein. The anti-angiogenic properties of THBS1 involves an upregulation of pro-apoptotic endothelial cell pathways and decreased vascular endothelial growth factor (VEGF) signaling.

Recent studies have demonstrated a relationship between TSP1 levels and TAAD development, and the *THBS1* gene has been localized as a potential therapeutic target for TAAD treatment. Yamashiro et al. demonstrated that upregulation of the *THBS1* gene contributed to the development of ascending aneurysm formation in vivo through the disruption of elastin-contractile units and dysregulation of the remodeling of the actin cytoskeleton [40]. This finding was particularly interesting as Rohrbach et al. demonstrated that the variants in *ZNF469* and *PRDM5* led to a significantly decreased production of the TSP1 protein product [6]. The TSP1 protein appears to be a biomarker of aortic disease, as TAADs have shown elevated levels of the protein [41]. These findings are valuable as they serve as a counter-argument for a potential protective role of the ZNF469 and PRDM5 genes in regard to TAAD formation. Regardless, this provides further support for further research into the role that these ECM regulatory genes play in TAAD formation.

## 5. PRDM5

### 5.1. PRDM5 and Association with Arterial Aneurysmal Diseases

Variants in *PRDM5* have shown to result in the downregulation of important ECM components, including fibrillar collagens (e.g., *COL4A1*), connective tissue components (e.g., HAPLN1, TSP1), and cell migratory and adhesion regulators (e.g., *EDIL3* and *TGFβ2*) in dermal fibroblasts [6,8]. Involvement in ECM regulation and maintenance has increased suspicion for a potential role in other diseases that are a result of variants in ECM-related genes, including TAAD.

Though prior findings related to *PRDM5* have been isolated to the development of BCS, we reported the first association between single-nucleotide variants (SNVs) in *PRDM5* and TAAD development [42]. Notably, two patients who presented with the presence of a TAAD had unique single-nucleotide variants p.R83H (c.248G > A) and p.E129A (c.386A > C) in PRDM5 [42]. Although it is not possible to state the precise probability of causation with these two variants, a Grantham score was calculated to demonstrate evolutionary distance and, thus, a prediction on the effects of amino acid substitutions. The Grantham score for the p.E129A was moderately radical (score of 107), suggesting further evolutionary distance and the potential for a greater effect. This is likely due to an acidic amino acid in glutamic acid being substituted for the neutral amino acid arginine [42]. Though the effects of these variants on downstream targets and tissue level effects have yet to be demonstrated, this represents the first association between *PRDM5* and aortopathy and, along with *ZNF469*, serves as another connection between ECM-related ocular disease and the development of aortopathy such as TAAD.

### 5.2. HAPLN1 as a Regulatory Target of PRDM5

Hyaluronan and proteoglycan link protein 1 (HAPLN1) is a protein encoded by the gene *HAPLN1* with diverse function, is thought to be a structural component of the ECM, and colocalizes with collagen in the ECM [43]. Burkitt Wright et al. demonstrated that when a loss-of-function variant was induced in *PRDM5*, resulting levels of HAPLN1 were decreased nearly 30-fold, suggesting a regulatory role of *PRDM5* in HAPLN1 expression [8]. HAPLN1 is thought to play a role in acute TAAD and dissection disease, as samples from such patients contained a significantly increased HAPLN1 concentration when compared not only to other tissues such as fat, skeletal muscle, and the left atrium but also to samples of the aorta in coronary bypass grafting patients [44]. HAPLN1′s role in the development of aortopathy has yet to be elucidated but, given its presence in disease aortas and relationship to PRDM5, collagens, and the ECM structure, it is a target gene that deserves attention in the pathogenesis of TAAD.

### 5.3. TGF-β2 as a Regulatory Target of PRDM5

Variants in *PRDM5* have resulted in the downregulation of many important ECM components in dermal fibroblasts, including *TGFβ2* [8]. *PRDM5*, a known player in BCS development, has recently been associated with TAAD development in two subjects as previously stated [42]. In addition to its association with LDS, the *TGFβ2* variant, reported by Boileau et al., has been reported as a driver of familial TAAD in two large family cohorts. The proposed mechanism of haploinsufficiency of the *TGFβ2* gene via frameshift and nonsense variants causes a lack of circulating cellular *TGFβ2* as a driver for the development of TAAD disease [45]. Though the mechanism behind *PRDM5* variant and subsequent aneurysm development is currently unknown, dysregulation in TGF-β signaling is an intriguing and potential mechanism due to its known role in aneurysm development.

## 6. Conclusions

**Figure 4:**
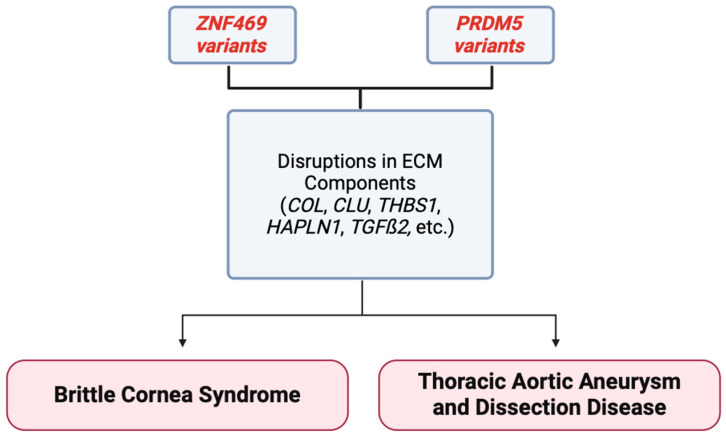
Summary figure demonstrating that variants in the *ZNF469* and *PRDM5* gene may cause disruption in ECM components. These disruptions have been correlated with the development of the ECM-related disease BCS in the past, and newer associations are being reported in thoracic aortic aneurysm and dissection formation. Variants in these genes are linking corneal and aortic diseases for the first time [17].

The ECM is the core structural support network of numerous tissues, including the eye and arterial vessels of the human anatomy. These two target genes provide a link between the eye and the aorta in terms of the structure and function of the ECM and the devastating clinical consequences of their dysfunction in both of these previously unrelated structures. This review highlights the phenotypic overlap between multiple syndromic diseases that were once thought to be independent, caused by variants previously not recognized as causal for aneurysmal and dissection disease. The driving force in this overlap is our hypothesis that downstream effects of variant forms of *ZNF469* and *PRDM5* genes on various collagens, clusterin, TSP1, TGF-β, and even further downstream effects on VSMC, elastin, and VEGF result in aberrant ECM proteins (Figure 4). These aberrant proteins have the potential to disrupt many processes of the ECM and, for the same reason that they cause BCS, have the potential to cause TAAD, and have phenotypic overlap with other disorders like EDS and osteogenesis imperfecta. 

## Data Availability

The data presented in this study are available on request from the corresponding author. The data are not publicly available due to patient privacy considerations.
